# Taxonomical composition and functional analysis of biofilms sampled from a nuclear storage pool

**DOI:** 10.3389/fmicb.2023.1148976

**Published:** 2023-04-13

**Authors:** Olivier Pible, Pauline Petit, Gérard Steinmetz, Corinne Rivasseau, Jean Armengaud

**Affiliations:** ^1^Université Paris-Saclay, CEA, INRAE, Département Médicaments et Technologies pour la Santé (DMTS), SPI, Bagnols-sur-Cèze, France; ^2^Université Grenoble Alpes, CEA, CNRS, IRIG, Grenoble, France; ^3^Université Paris-Saclay, CEA, CNRS, Institute for Integrative Biology of the Cell (I2BC), Gif-sur-Yvette, France

**Keywords:** biofilm, industrial pool, microorganisms, metaproteomics, taxonomy, detection, proteotyping, pan-proteomics

## Abstract

Sampling small amounts of biofilm from harsh environments such as the biofilm present on the walls of a radioactive material storage pool offers few analytical options if taxonomic characterization and estimation of the different biomass contributions are the objectives. Although 16S/18S rRNA amplification on extracted DNA and sequencing is the most widely applied method, its reliability in terms of quantitation has been questioned as yields can be species-dependent. Here, we propose a tandem-mass spectrometry proteotyping approach consisting of acquiring peptide data and interpreting then against a generalist database without any *a priori*. The peptide sequence information is transformed into useful taxonomical information that allows to obtain the different biomass contributions at different taxonomical ranks. This new methodology is applied for the first time to analyze the composition of biofilms from minute quantities of material collected from a pool used to store radioactive sources in a nuclear facility. For these biofilms, we report the identification of three genera, namely *Sphingomonas*, *Caulobacter*, and *Acidovorax*, and their functional characterization by metaproteomics which shows that these organisms are metabolic active. Differential expression of Gene Ontology GOslim terms between the two main microorganisms highlights their metabolic specialization.

## Introduction

Some microorganisms can subsist and even flourish in extreme conditions in which most multicellular organisms, like animals or humans, cannot survive. Bacteria, fungi, archaea, and micro-algae have indeed been found in acidic or alkaline media, in environments with extremely low or high temperature, high pressures, or high salt concentration. Some of these organisms are known to withstand high radiation doses, such as the bacterium *Deinococcus deserti* isolated from the Sahara desert (de Groot et al., 2005), the archaeon *Thermococcus gammatolerans* screened from a deep-sea hydrothermal vent (Jolivet et al., 2003), or the green algae *Coccomyxa actinabiotis* isolated from a pool used to store spent nuclear fuels (Rivasseau et al., 2013, 2016). Numerous studies have focused on deciphering the molecular mechanisms explaining the remarkable tolerance properties of these model organisms (de Groot et al., 2009; Zivanovic et al., 2009; Baudet et al., 2010), *in situ* environmental studies of such microorganisms are relatively scarce. Noteworthy, several bacteria belonging to *Burkholderia* and *Ralstonia* genera have been identified in a French storage pool (Gales et al., 2004), *Afipia, Chriseobacterium, Nocardia, Pseudomonas*, and *Stenotrophomonas* genera in the cooling pool of a Spanish nuclear power plant (Chicote et al., 2005), *Pelomonas* and *Methylobacterium* genera in the water basin surrounding a nuclear research reactor vessel in Belgium (Van Eesbeeck et al., 2021). More surprisingly due to very high levels of irradiation, the genera *Variovorax* and *Sphingomonas* have been identified in the core cooling pool of an operating nuclear reactor in France (Petit et al., 2020).

In nature, many microorganisms can form biofilms to colonize a specific environment and facilitate their long-term fate. For this purpose, consortia of microorganisms usually adhere to a surface, are embedded within a self-produced matrix of extracellular polymeric substances and can further colonize their surroundings (Eigentler et al., 2022). This specific growth pattern is actually more efficient than the planktonic form, because it protects against external conditions and their associated changes. Inside a biofilm, the pH can be modified and maintained at an optimal level, and nutrients and organic material can be recycled more easily, creating more favorable conditions for the survival and growth of microorganisms (Jefferson, 2004). The advantages obtained by growing as biofilms may explain the fact that in aquatic environment less than 0.1% of the bacteria are under planktonic form (Costerton et al., 1995). Indeed, microbial biofilms have been detected in very inhospitable environments such as spent nuclear fuel pools (Gales et al., 2004; Sarro et al., 2005), but their exact composition and functioning have not been established (Chicote et al., 2005; Rivasseau et al., 2013).

Assessing the microbial diversity of aquatic environments is usually done by extracting the DNA content from the sample, followed by amplifying 16 or 18S rRNA gene, sequencing the amplicons and comparing sequences with reference databases (Esposito and Kirschberg, 2014). This approach has been challenged because the number of copies of the 16S rRNA gene per chromosome and the number of copies of the chromosomes in bacteria can vary depending on the strain and its physiological state (Tourova, 2003; Vetrovsky and Baldrian, 2013). Indeed, the number of copies of rRNA gene can vary from one to up to 15 copies per cell. Moreover, the DNA extraction may be not strictly identical depending on the microorganisms present and the type of biofilms to be analyzed. Last, the amplification of 16S rRNA gene of archaea or bacteria and the amplification of 18S rRNA gene of eukaryotes are performed with different primer sets, so that the whole taxonomic panorama cannot be compared in terms of biomass quantities. Furthermore, within the same taxonomic group, neighboring sequences of the 16/18S rRNA locus may differ slightly and influence the amplification rate. For all these reasons, amplification may not be exactly the same for different strains and phyla present in the sample. Alternatives for taxonomy can be based on cataloging of genomic sequences (metagenomics) or protein sequences (proteotyping). While metagenomics provides an insight into the potential of the microorganisms present in a sample, metaproteomics is dedicated to the global study of the proteins present in a complex sample comprising numerous different organisms in order to better understand the key pathways and the physiology of the main players, and to study the interactions of a microbial community with its environment (Wilmes and Bond, 2006). Interestingly, the approach based on tandem mass spectrometry of complex peptide mixtures provides per principle two distinct but complementary pieces of information: the taxonomic characterization of the organisms present and the identification and quantification of the proteins in the system, thus describing the functioning of the system (Armengaud, 2023).

Metaproteomics has been used to get new insights into a natural acid mine drainage microbial biofilm (Ram et al., 2005), showing that the dominant bacteria belonged to the *Leptospirillum* genus. The experimental data covered most of the proteins from this dominant organism, as well as the four other most abundant species in the biofilm. Proteins involved in protein refolding and in response to oxidative stress appeared to be highly synthesized in such biological material, suggesting that damage to biomolecules is a key challenge for survival to the harsh conditions encountered by the biofilm, i.e., extreme acidity and high toxic metal concentrations. These investigations also provided insights into the functioning of these organisms as consortia, highlighting how essential functions such as nitrogen fixation and biofilm polymer production are distributed among members of the community. Last, a highly abundant protein with a previously unknown function was found to be an iron-oxidizing cytochrome, a key enzyme for energy production in these biofilms. The analysis of this specific microbial biofilm was further investigated by comparative proteogenomics. This revealed how genomic variation within closely related bacteria could contribute to their ecological divergence (Denef et al., 2010). Other studies of environmental biofilm communities have focused on marine biofilms sampled from destroyer hulls (Leary et al., 2014) and wastewater treatment biofilms (Abram et al., 2009), leading to a taxonomic classification of the major components of the microbial communities and the description of the metabolic activities of key microorganisms.

The most common strategy for conducting a metaproteomic study is to first collect metagenomic data on the same sample in order to obtain a sample-specific protein sequence database. This can be done either by performing a systematic six-frame translation of the reads or contigs or by their tentative annotation, resulting in an inflated proteogenomics protein sequence database (Blakeley-Ruiz and Kleiner, 2022). Subsequently, MS/MS spectra recorded from peptides generated by proteolysis of the extracted proteins are assigned according to this metagenomics information. However, for precious samples obtained in small quantities, or for faster analysis, obtaining this metagenomic information can be far too complex and alternatives based on the use of generic database have proved valuable even for complex samples such as sediments (Jouffret et al., 2021).

In this work, we collected minute amounts of biofilms from an industrial facility and applied such a new proteotyping approach consisting in the acquisition and interpretation of metaproteomic data against a generalist database. The assigned peptides were then analyzed to get insight into the taxonomy of the organisms present in the sample. This method is being used for the first time to decipher the composition of biofilms harvested from a pool used to store radioactive sources in a nuclear facility, and understand the metabolism of the main microbial components. A method for recovering a few milligrams of biological material from this hostile environment, compatible with proteotyping of mixtures of microorganisms, is also proposed.

## Materials and methods

### Industrial facility studied

The biofilm samples were collected from a 5.5 m deep pool used for storage of radioactive sources and irradiation experiments at CEA, Saclay, France. The pool contained water purified by reverse osmosis. Its conductivity, pH, and temperature were 127 μS/cm, pH 7.8 and 26°C, respectively. Analysis of the main anions and cations revealed the presence of chloride, sodium, potassium, calcium, and magnesium at average concentrations of 2.2, 5.8, 1.6, 41, and 1.7 mg/L, respectively. Nitrates, phosphates, and sulfates were below the quantification limit of 5, 0.2, and 10 mg/L, respectively. No radionuclides were detected. The water was filtered daily for 1 h on 0.5 μm filters. When irradiation sources were present in the pool, the dose rate on the wall where the biofilms were collected reached 100 Gy/h. Dose rates were assessed by direct dosimetry measurement using a UNIDOS dosemeter (PTW) and dose rate received on the walls were estimated taking into account water column attenuation.

### Biofilm collection

Biofilms were sampled directly from the pool walls at different locations (specified in the Results section) using sterile Spontex scraping sponges (Spontex-Mappa). For each sample, a sponge was taped to a metal pole and was then used to scrub the wall underwater. After scrubbing an area of approximatively 100 cm^2^, for less than 1 min, the pole was slowly raised, the tape removed and the sponge placed in a sterile hermetic plastic bag for transport. In the analytical laboratory, each sponge containing the biofilm was introduced inside a sterile flask containing 100 ml of sterile water. To suspend the biofilm, the sponge was stirred with a sterile spatula and the flask was shaken vigorously to detach the biological material from the sponge fibers. After removing the sponge, the resulting liquid was centrifuged at 15,000 *g* for 20 min at room temperature to pellet the biological material. All but 1 ml of the supernatant was discarded and the pellet was dissolved in this 1 ml volume. For metaproteomics analysis, a 750 μl volume of the suspension was stored at −20°C until metaproteomic analysis.

### Protein extraction and trypsin proteolysis

After thawing the sample tubes on ice, the samples were introduced into a 2 ml Precellys tube (Bertin Technologies) containing 200 mg of glass beads as recommended (Hayoun et al., 2019). The samples were subjected to three cycles of bead beating at 6,500 rpm for 20 s using a Precellys grinder (Bertin Technologies). After grinding, the extracted proteins were precipitated by adding a volume of trichloroacetic acid solution prepared at 50% in water to obtain a final acid concentration of 10%. The sample was then centrifuged at 16,000 *g* for 10 min. The resulting pellet was dissolved in 25 μl of LDS 1× Laemmli buffer (Invitrogen) consisting of 106 mM Tris/HCl, 141 mM Tris base, 2% lithium dodecyl sulfate, 10% glycerol, 0.51 mM EDTA, 0.22 mM SERVA Blue G250, 0.175 mM phenol red, buffered to pH 8.5 and supplemented with 2.5% beta-mercaptoethanol. The sample was heated at 99°C for 5 min, followed by treatment in an ultrasonic bath for 5 min. The sample was then loaded onto a 4–12% gradient 10-well NuPAGE gel (Invitrogen). Proteins were subjected to electrophoresis for 5 min as previously described (Hartmann et al., 2014). After staining with Coomassie Blue Safe (Invitrogen), the polyacrylamide band corresponding to the whole proteome was sliced. The polyacrylamide band was then destained with ultra-pure water, reduced with dithiothreitol and treated with iodoacetamide, before proteolysis with Trypsin Gold Mass Spectrometry Grade (Promega) in the presence of 0.01% ProteaseMAX surfactant (Promega), as previously described (Rubiano-Labrador et al., 2014).

### NanoLC-MS/MS analysis

The extracted peptides (10 μl) were analyzed with a Q-Exactive HF mass spectrometer (Thermo) equipped with an ultra-high field Orbitrap analyser and coupled to an Ultimate 3000 176 RSL Nano LC System (ThermoFisher). This analysis was performed in data-dependent mode in similar conditions as those previously described (Grenga et al., 2020), i.e., fragmentation and MS/MS spectra acquisition was done on specific peptide ions identified by their molecular masses divided by their electric charge. Peptides were injected onto a reverse phase Acclaim PepMap 100 C18 column (3 μm, 100 Å, 75 μm id × 500 mm) and resolved at a flow rate of 0.2 μl/min with a 60 min gradient of CH_3_CN in presence of 0.1% formic acid. The gradient during MS/MS acquisition consisted in 50 min from 2 to 20% CH_3_CN, followed by 10 min from 20 to 32% CH_3_CN. A quick wash of the column was done with an additional gradient from 32 to 72% CH_3_CN in 1 min, before equilibrating the column with 2% CH_3_CN. The Q-Exactive HF instrument was operated with a Top20 strategy: full scan mass spectra were acquired from *m/z* 350 to 1,500 with an Automatic Gain Control Target and resolution set at 3 × 10^6^ ions and 60,000, respectively, for the MS scan, and 1 × 10^5^ ions and 15,000, respectively, for the MS/MS scan. MS/MS scan acquisition was started when the intensity threshold of 170,000 was reached. The maximum fill time was 60 ms and the isolation window for the quadrupole was 1.6 *m/z*. Only ions with potential charge states of 2+ and 3+ were selected for MS/MS with a dynamic exclusion time of 10 s.

### Proteotyping MS/MS data interpretation

The proteotyping interpretation of MS/MS spectra followed the same procedure as described previously (Grenga et al., 2022). The version of the National Center for Biotechnology Information non-redundant (NCBInr) database used includes 76,068,736 protein sequence entries totaling 27,658,295,194 amino acids. For peptide inference, MS/MS spectra were searched using the MASCOT 2.2.04 search engine (Matrix Science, London, UK). Peptides were assigned to MS/MS spectra using Mascot with the following parameters: full trypsin specificity, maximum one missed cleavage, mass tolerances of 5 ppm on the parent ion and 0.02 Da on the MS/MS, static modification of carboxyamidomethylated cysteine (+57.0215) and oxidized methionine (+15.9949) as dynamic modification. Post-processing of Mascot dat files was done as follows using Python version 2.6.6 taxonomy identification procedures. Mascot DAT files were parsed using the Python version of Matrix Science msparser version 2.5.1 with the ms_peptidesummary function.^1^ Peptide-Spectrum Matches (PSMs) were validated with a Mascot expectation value below 0.05 using Mascot identity threshold (MIT), and by allowing multiple PSMs per MS/MS spectrum for ion scores higher than 98% of the top ion score. Peptides associated to spectra were assigned to taxa, resulting in Taxon-Spectrum Matches (TSMs) at each possible taxonomic level, species, genus, family, order, class, phylum, and superkingdom, as previously defined (Pible et al., 2020). For each taxon at each level, an identification of matching peptide sequences and TSMs was performed, as well as a count of specific peptide sequences and corresponding specific PSMs. The taxonomical ranks considered in this study are: superkingdom, phylum, class, order, family, genus, species, and subspecies, forming lineages starting at the top taxon Bacteria-Eukarya-Virus-Archaea (BEVA) and going down the list of ranks. At each taxonomic level, children taxa of already validated parent taxa (BEVA is pre-validated) were assessed using the following thresholds: 7 specific peptides, or max (#totalTSMs/100, 30) added TSMs (thresholdTSM).

At each taxonomical rank, the pertaining taxa were ranked by the number of specific peptides, from highest to lowest. The top taxon per parent was compared to the specific peptides threshold, cycling through parent taxa. If a validated parent had no validated subtaxon, the child with the maximum number of TSMs was validated as the best representative of the clade. TSMs corresponding to validated taxa were added to a pool of TSMs at the taxonomical rank (RankValTSMs). This was reiterated for the next taxa in order, one per parent, until no more taxa with sufficient specific peptides could be found. The subtaxa were then reordered by the number of corresponding TSMs, excluding RankValTSMs, and compared to the threshold TSM, cycling through the parent taxa. The process was ended when the number of RankValTSMs (rank) was above 0.992 × RankValTSMs (parents), or when no additional taxon could meet validation criteria. The conjunction of a Mascot MIT *p*-value of 0.05, which is very stringent for large databases such as NCBInr, and of high thresholds (7 specific peptides to validate a taxon on a large database and reduced MS/MS data sets is very drastic) contributes to the selection of high-confidence taxa, at the expense of sensitivity.

### Metaproteomics interpretation

For the metaproteomic interpretation of the MS/MS spectra, the database used was the merge of all protein sequences of *Sphingomonas*, *Caulobacter*, and *Acidovorax* contained in the NCBInr fasta file, as well as the most common proteomics contaminants (MaxQuant contaminants.fasta database). The *Sphingomonas-Caulobacter-Acidovorax* pan-proteomic database comprises 498,167 protein sequence entries totaling 163,630,882 amino acids from 4,032 different annotated genomes. Peptide-to-MS/MS spectrum matches (PSMs) with a MASCOT expectation *p*-value below 0.002612 were selected. This *p*-value threshold was obtained by searching the merged data set on the forward and reverse database as a decoy, and adjusting the *p*-value to correspond to a false discovery rate of 1%. Proteins were grouped on the basis of their shared peptides. Protein abundances were assessed by their spectral counts. Gene Ontology (GO) annotations of proteins were obtained using DIAMOND (Buchfink et al., 2015) to search for protein sequences against the Swiss-Prot database. Hits with more than 30% identity were retained, which indicates proteins of similar tertiary structures, and allows retrieval of most of the closest proteins with GO annotation as previously demonstrated in the first critical assessment of protein function annotation (CAFA) experiment (Radivojac et al., 2013). The Gene Ontology Annotation (GOA) database^2^ was used to map Swiss-Prot entries with GO terms. However, given the poor fine-grained results of most approaches, including the reference BLAST approach in the CAFA experiment, we report GO ontologies at a higher level in the GO tree using GOslim terms. This GO subset mapping was processed using the Map2Slim option in OWLTools^3^ and the generic GOslim list of GO entries.^4^

### MS/MS dataset

The mass spectrometry proteomics data have been deposited to the ProteomeXchange Consortium via the PRIDE partner repository with the dataset identifier PXD039499 and http://www.ebi.ac.uk/pride/archive/projects/PXD039499.

## Results

### Biofilm sampling in the CEA-Saclay nuclear facility

**FIGURE 1:**
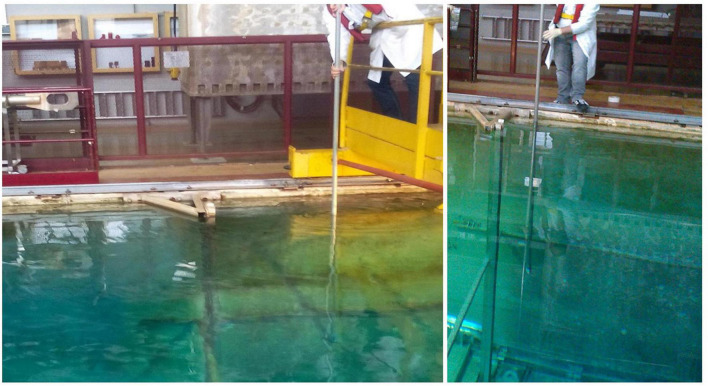
Surface of the water pool that was sampled. The pictures show the biofilm sampling on the north wall using the pole with the scraper sponge attached (credit: Corinne Rivasseau).

We proposed a direct sampling of the biofilm on the walls of a pool used to store nuclear elements. In such extreme environment, it appears difficult to introduce an experimental support and allow biofilms to grow for several weeks or months. Furthermore, such a biofilm may not be representative of the biological deposit on the walls as the support may be different. The pool chosen for this case study did not contain any radionuclides, allowing direct manipulation of the collected biofilms for full characterization with omics tools. The selected facility is used to carry out irradiation experiments with cobalt irradiation sources positioned either in a concrete bunker or in the pool next to the bottom. During storage, the sources are maintained under water. Figure 1 shows a photograph of the surface of the pool in contact with the air. The water in the pool is oligotrophic and slightly alkaline. It contains very few nutrients, the main macronutrients such as nitrate, phosphate, and sulfate being undetectable. In the pool, the sources are positioned at a specific location, a first set near the bottom of the north wall and a second set near the bottom of the west wall. Therefore, the bottom of the north and west walls are intermittently exposed to gamma radiation. The west wall was estimated to be exposed at a dose rate of 100 Gy/h in front of the sources. One meter below the surface, the radiation dose rate is zero. Five months before sampling, the bottom of the north wall was exposed to a radiation dose of 12–24 kGy. In the three weeks before sampling, the bottom of the west wall was irradiated for a cumulative time of 425 h and the sources were still in place at the time of sampling. We collected a first biofilm sample, called Bottom Western Wall (BWW) biofilm, on the west wall 4 m below the water surface, just above the top of the sources. The dose rate received by the biofilm at this location is estimated to be 1 Gy/h, giving a cumulative dose of about 400 Gy in the three weeks prior to sampling. Another biofilm was collected near the water surface on this wall (1 m deep) and called Top Western Wall (TWW) biofilm. Two other biofilms were collected further from the sources on the northern wall, one at 4 m depth and called Bottom Northern Wall (BNW) biofilm and the other near the surface (1 m deep) and called Top Northern Wall (TNW) biofilm.

Most studies of biofilms in aquatic environments to date have been carried out either by sampling directly at the air/water interface or after removing the biofilm support from the water and scraping the biofilm with a spatula (Gomez-Alvarez et al., 2012; Leary et al., 2012). Here, we proposed to detach the biofilm from the wall directly underwater using a sponge rather than a spatula to facilitate sample recovery. We chose to use a Spontex abrasive sponge made of synthetic fibers chemically bonded together by a resin. Sampling 4 m below the surface of the water required the use of a sponge attached to a pole by adhesive tape. This scrubbing sponge allowed easy handling several meters under water, as the sponge fibers trapped some of the biofilm and allowed it to be recovered. After rubbing the wall, the sponge was recovered and part of the biofilm could be directly observed thanks to a slight yellowish color and recovered for analysis.

### Proteotyping the microbial composition of biofilms by phylopeptidomics

**FIGURE 2:**
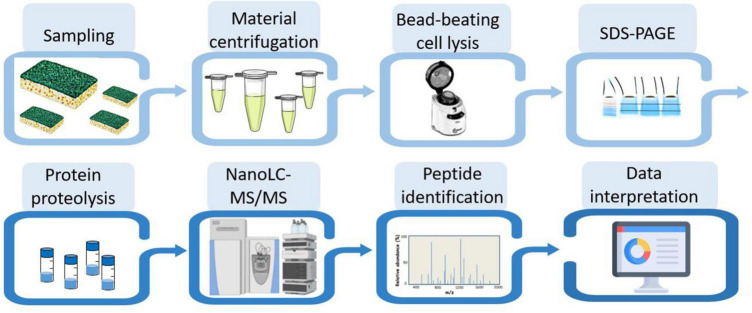
Experimental workflow for metaproteomic analysis. The eight main steps applied to the four biofilm samples are symbolized.

Figure 2 shows the experimental workflow used to identify the taxa present in the different samples: protein extraction, peptide analysis by tandem mass spectrometry coupled to reverse phase chromatography, and metaproteomics analysis of the acquired data. Due to the scarcity of biological material recovered from the sponge, only one analytical run could be performed per sample. To proteotype the microbial composition of the biofilm, MS/MS spectra were interpreted against the NCBInr database, a generalist database comprising the annotated proteomes of several tens of thousands of reference organisms. The identified peptide sequences were then mapped to taxa whose proteomes contained these sequences. Taxa-specific peptides were those specifically associated with a given taxonomic unit, taking into account all different taxonomical ranks. The number of peptide sequences specific to a taxonomic branch, i.e., taxon-specific peptide sequences, logically guarantees the presence of this taxonomic branch in the sample. In addition, the TSMs value is a complementary piece of information that can be used to assess the abundance of each of the identified taxa. The proteotyping capability of the method applied here is based on the validation of the taxa, from the superkingdom taxonomic level down to the species level, using both corresponding specific peptides and TSMs attributed to taxa.

Table 1 shows the number of MS/MS spectra recorded per sample. It ranges from 4,657 (TNW) to 25,777 (BWW). These numbers are directly correlated to the quantity of protein material recovered per sample. Roughly, they correspond to the equivalent of 0.02 and 0.1 mg of biological material as other experiments performed with a pure microbial culture under the same analytical conditions yielded ≈50,000 MS/MS spectra for 1.9 mg of wet biological material when one tenth of the peptides produced were injected into the mass spectrometer. The TNW biofilm was the less abundant in terms of extracted proteins. However, this data set, acquired within 1 h of tandem mass spectrometry, is still relatively rich in information as a total of 594 peptide sequences could be assigned. The percentage of MS/MS spectra assigned per sample varies between 10% (BWW) and 16% (TNW) as shown in Table 1. This number illustrates the conjunction of two factors: an expected lack of completeness of the generalist database used for the interpretation on the one hand, and a possible decrease in the quality of some MS/MS spectra due to the co-elution of diverse peptide entities on the other hand. As recently pointed out, the diversity of peptides in metaproteomic samples can be huge, as multiple strains of the same species can cohabit (Armengaud, 2023). However, these percentages compared favorably with other proteomics search results on non-model organisms whose genome has not yet been sequenced and appropriately annotated (Trapp et al., 2016; Gouveia et al., 2020) or for metaproteomic studies of environmental samples such as soils (Jouffret et al., 2021; Lidbury et al., 2022).

**TABLE 1:** Data (or characteristics) of the MS/MS spectra acquired per LC-MS/MS run and their interpretation, for each biofilm.

	Biofilm			
	**BNW**	**TNW**	**BWW**	**TWW**
# MS/MS spectra	14,677	4,657	25,777	16,052
# TSMs All taxa	2,095	728	2,645	1,744
# peptide sequences	1,387	594	1,931	1,325
% assignment	14.3	15.6	10.3	10.9

#### Identifying the microorganisms present in the BNW biofilm as a case study

Table 2 reports the numbers of TSMs and specific peptides of the validated taxa at the different possible taxonomical ranks for the four samples. For the BNW biofilm, a total of 1,168 TSMs and 724 unique taxon-specific peptide sequences were exclusive of the Bacteria superkindgdom. Among these, the vast majority are exclusive to a single validated phylum: Proteobacteria, with 1,136 TSMs and 580 unique phylum-specific peptide sequences. We noted that the decrease in terms of unique taxon-specific peptide sequences (−20%) is sharper than the decrease in terms of TSMs (−3%) when moving from the superkingdom to the phylum taxonomical rank. This discrepancy arises from peptide sequences belonging to highly conserved parts of housekeeping proteins systematically present in bacteria. While these peptides are not considered as specific of the Proteobacteria phylum, they contribute to the bacterial signal. Because no other phylum is represented in the data set (i.e., validated with sufficient specific peptides or added TSMs), the whole specific bacterial signal can be then assigned to the Proteobacteria. The same reasoning can be applied at a lower taxonomical rank. At the Class rank, two children taxa could be validated: Alphaproteobacteria and *Betaproteobacteria* with 493 and 55 unique taxon-specific peptides, respectively. The corresponding quantities of the two classes could be roughly assessed through their respective TSMs: 1,011 and 171, respectively. The fraction of peptides shared between Alphaproteobacteria and Betaproteobacteria is very small as can be estimated by comparing the sum (1,011 + 171) and 1,136 at the upper level, i.e., 4% of the TSMs are shared between both classes. Fractions based on specific peptides would estimate Betaproteobacteria at 55/580 = 9.5% of the bacterial content, while their quantification based on TSMs would be 171/1136 = 15%, which is more accurate in the case of low peptide overlap between clades. At the Order rank, the Betaproteobacteria taxon was explained by the presence of a unique order: Burkholderiales with 47 unique peptides and 160 TSMs. The Alphaproteobacteria taxon signal is subdivided into two children orders: Sphingomonadales and Caulobacterales. More taxonomic precision could be achieved at the Family and Genus levels. A unique genus and family could be delineated per order by phylopeptidomics. Finally, the *Sphingomonas* (Sphingomonadaceae), *Caulobacter* (Caulobacteraceae), and *Acidovorax* (Comamonadaceae) genera were validated with 340, 33, and 16 unique taxon-specific peptides, respectively. Their respective quantities may be roughly assessed from their TSM signal: 80% (898), 11% (122) and 9% (96). The proportion of each genus would be significantly different if based on the number of taxon-specific peptides: 87% (340), 8% (33), and 4% (16). Indeed, the number of taxon-specific specific peptides can vary significantly due to the phylogenetic relationships between the organisms present in the sample and the density of sequenced genomes per phylogenetic branch. Therefore, it is not a reliable parameter for quantifying the ratio of organisms as discussed previously (Pible et al., 2020).

**TABLE 2:** Number of taxa-to-spectrum matches (TSMs) and taxon-specific peptides (spePEPs) detected at the different taxonomical ranks in the four biofilms*.

Taxonomy		BNW		TNW		BWW		TWW	
**Rank**	**Taxa**	**# TSMs**	**# spePEPs**	**# TSMs**	**# spePEPs**	**# TSMs**	**# spePEPs**	**# TSMs**	**# spePEPs**
Superkingdom	Bacteria	1,168	724	577	427	1313	913	676	484
Phylum	Proteobacteria	1,136	580	572	326	1264	743	634	367
Class	Alphaproteobacteria	1,011	493	547	303	1205	703	551	316
	Betaproteobacteria	171	55	42	9	n.d.	n.d.	99	24
Order	Sphingomonadales	903	393	523	264	1142	589	484	1134
	Caulobacterales	148	39	51	14	87	10	70	1464
	Burkholderiales	160	47	33	9	n.d.	n.d.	90	1178
Family	Sphingomonadaceae	903	383	523	259	1140	568	484	255
	Caulobacteraceae	148	39	51	14	87	10	70	22
	Comamonadaceae	119	29	20	3	n.d.	n.d.	52	13
Genus	*Sphingomonas*	893	340	521	226	1131	497	478	214
	*Caulobacter*	122	33	35	9	52	7	53	18
	*Acidovorax*	96	16	16	3	n.d.	n.d.	40	8

*If a validated parent had no validated subtaxon, then the child with the maximum number of TSMs was validated as the best clade representative (gray background). n.d., not detected (below thresholds).

While the decrease in the number of specific peptides is rather sharp along the taxonomical ranks considered (from 724 at the Superkingdom taxonomic rank to 389 at the Genus taxonomical rank), the TSMs parameter is rather stable (from 1,168 to 1,111, respectively). Thus, no other main contributor to the biofilm, such as an unsequenced branch of life of bacteria, should be considered to explain the proteomic signal. The identification of organisms at a lower taxonomic rank than the genus rank would require a larger experimental dataset, thus more starting material, and a more comprehensive database for the three genera identified. Because the biofilm may comprise several closely related species whose genome have not yet been sequenced and reported in the NCBInr database, the identification at the species level in this study is only speculative. The most closely related major species identified by their specific peptides were: *Sphingomonas sp. Leaf257* (TaxID 1736309), *Sphingomonas parapaucimobilis* (TaxID 28213), *Sphingomonas melonis* (TaxID 152682), *Caulobacter vibrioides* (TaxID 155892) and *Acidovorax sp. CF316* (TaxID 1144317). As the number of bacteria whose genome has been sequenced and whose species taxonomy is well established in these important groups of environmental bacteria is still rather low (i.e., 45 for *Sphingomonas*, 5 for *Caulobacter*, and 17 for *Acidovorax*), we cannot ascertain at the present stage the accuracy of taxa identified at the species taxonomical rank. For example, either the presence of a strain with intermediate genome characteristics between *Sphingomonas sp. Leaf257*, *S. parapaucimobilis*, and *S. melonis*, or the mixture of three closely related species could explain the *Sphingomonadales* signal.

#### The biofilm composition is relatively similar at three sampling points

Table 2 presents also the phylopeptidomics data for the other three biofilms. For the TNW biofilm, a low quantity of material was noted from the number of MS/MS spectra recorded (4,657 compared to 14,677 for BNW). Although this decrease is important (one third of BNW), the phylopeptidomics signal could be confidently assigned to the genus level as previously described for the BNW biofilm. Here, the presence of three orders was validated: Sphingomonadales, Caulobacterales, and Burkholderiales, with 264, 14 and 9 unique specific peptides, respectively. At the Family taxonomic rank, sufficient signal could certify the presence of Sphingomonadaceae (259 taxon-specific peptides) and Caulobacteraceae (14 taxon-specific peptides), but not of Comamonadaceae (only 3 taxon-specific peptides). Logically, the same occurs at the Genus taxonomical rank, with 226, 9 and 3 taxon-specific peptides for the presence of *Sphingomonas*, *Caulobacter*, and *Acidovorax*, respectively. Regarding the TWW biofilm, a large quantity of MS/MS spectra was recorded. In this case, a rather low number of TSMs was assigned to the superkingdom Bacteria (676) compared to the BNW biofilm (1168). Anyway, the three genera present in this sample were unambiguously identified by phylopeptidomics: *Sphingomonas*, *Caulobacter* and *Acidovorax*, certified by 214, 18 and 8 taxon-specific peptides, respectively. This shows the relative homogeneity of the biofilms in the pool, with the exception of the BWW biofilm as presented below.

#### The BWW biofilm stands out from the others

The latest biofilm sample, BWW, provided the largest MS/MS spectra data set, i.e., corresponding to the most material-rich sample. In this case, only one class was validated (*Alphaproteobacteria*), with a large number of taxon-specific peptides (703). The *Betaproteobacteria* class counts only three specific peptides, which is below the validation threshold. Two orders, Sphingomonadales and Caulobacterales, contributed to this signal. The genera identified were: *Sphingomonas* and *Caulobacter*, with 497 and 7 taxon-specific peptides, respectively. Therefore, the BWW biofilm stands out from the others as no evidence of *Acidovorax* could be detected in this biofilm although a larger amount of material was available. At the species taxonomical rank, the most-closely related species identified by their specific peptides in all the sampled biofilms were the same as those identified for the BNW biofilm, with the exception of BWW, which does not comprise any detectable *Acidovorax* signal. Correlating these results with the radiation doses received by the different biofilms, irradiation of the north wall five months before sampling had no further effect on the microbial community in the BNW biofilm. In contrast, irradiation of the west wall in the three weeks prior to sampling could explain *Acidovorax* disappearance from the BWW biofilm community.

**FIGURE 3:**
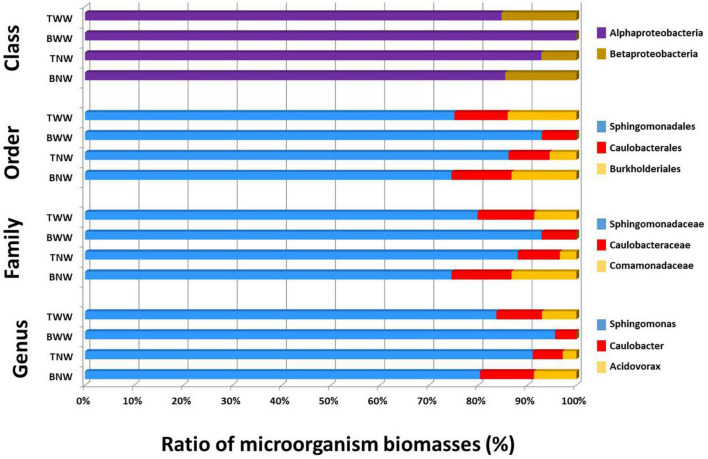
Proportion of each genus in the four biofilms after phylopeptidomic analysis. Biofilms were recovered from four locations (TWW, BWW, TNW, and BNW). The ratios of microorganisms were established either by Class-specific TSMs, Order-specific TSMs, Family-specific TSMs or Genus-specific TSMs for the four samples.

Figure 3 shows the ratio of microorganisms assessed for the four biofilms sampled based on the TSMs criterion at four taxonomic ranks, namely Genus, Family, Order, and Class. In all four biofilms, *Sphingomonas* was the main genus contributor. *Sphingomonas* and *Caulobacter* were systematically found. *Acidovorax* was another genus detectable in BNW and TWW, as well as faintly perceived in TNW (formerly identified only by three taxon-specific peptides) but explaining a Burkholderiales MS/MS-certified signal (nine taxon-specific peptides). The BWW biofilm signal differed from the others due to the absence of Betaproteobacteria phylopeptidomics signal. Some slight discrepancies in terms of relative ratio of each genus were noted between the different taxonomic ranks (Figure 3). However, because the higher taxonomical ranks benefit from a greater number of genome-sequenced microorganisms in the database than the lower taxonomic ranks and thus from a greater number of protein sequences, their reliability in terms of quantitation should be better.

#### Metaproteomics insights into the *Sphingomonas-Caulobacter-Acidovorax* consortium

Although the signal was relatively low, metaproteomics interpretation could be achieved using a reduced pan-proteomics database encompassing the protein sequences of all genome-wide sequenced strains belonging to the three genera detected in the samples and usual contaminants in proteomics experiments. This pan-proteomic database included a total of 498,167 protein sequences from 4,032 different annotated genomes. By merging the MS/MS datasets from the four biofilms, a total of 3,165 peptides were identified and 695 proteins were validated (Supplementary Tables 1, 2). Based on the shared peptide sequences, these proteins were grouped into 583 protein groups. Evidently, most of the detected proteins were from *Sphingomonas* (490), while proteins from *Acidovorax* and *Caulobacter* were less detected (63 and 81, respectively). The remaining 60 proteins were attributed to contaminants such as keratins. The numbers of protein groups identified in the four biofilms were relatively different: 340 (BNW), 173 (TNW), 388 (BWW), and 217 (TWW), respectively. In total, 71 protein groups were common to all four samples. Within this subset, the most common bacterial proteins were the elongation factors Tu and G involved in translation, the molecular chaperone GroEL, the subunit beta of DNA-directed RNA polymerase, and both the alpha and beta subunits of ATP synthase. This suggests that the cells from the three genera were metabolically active.

Among the most abundant proteins identified for *Sphingomonas* were the flagellar motor proteins MotB and MotA, several TonB-dependent transporters (x8), several catalases (x8), several ribosomal proteins (L3, L9, L11, L13, L19, L23, L28, S2, S5, S6, and S7), the cell division protein FtsZ, and several enzymes of the central metabolism (alcohol dehydrogenase, acyl-CoA dehydrogenase, aconitate hydratase, adenosylhomocysteinase, isocitrate dehydrogenase, adenylosuccinate lyase, dihydrolipoamide dehydrogenase, malate synthase). Several TonB-dependent transporters (x3) and the two flagellar motor proteins MotB and MotA were also identified for *Caulobacter*, as well as two subunits of the nitrate reductase A (subunit alpha and subunit beta). Specific proteins were identified in *Acidovorax* such as porins (x4), glutamate dehydrogenases (x2), outer membrane protein W, outer membrane protein/peptidoglycan-associated lipoprotein, alkyl hydroperoxide reductase, and glutamine synthetase. Interestingly, several proteins potentially linked to DNA repair were observed in *Sphingomonas* (UvrD-helicase known to act on double strand DNA with a 3’-single strand DNA tail, the DNA recombination/repair protein RecA, which catalyzes ATP-dependent DNA strand exchange reaction for repair of DNA double-strand breaks by homologous recombination, and the replicative DNA helicase, which unwinds the DNA duplex at the replication fork of the chromosome) and *Caulobacter* (RmB DNA/RNA helicase). Furthermore, two DNA-binding response regulators (OmpR and CitB–like transcription regulator proteins) have been identified in *Sphingomonas*. In several microorganisms, the SOS regulon encodes proteins involved in the coordinated response to DNA damage (Maslowska et al., 2019). Further molecular biology experiments would be needed to gain a better insight into the regulation of the corresponding genes.

A functional analysis of the gene ontology was performed: Supplementary Table 2 shows the results at the protein information level and Supplementary Tables 3, 4 display the data from the enriched functional analysis. Supplementary Table 3 includes results from the irradiated zone BWW, while Supplementary Table 4 only considers the 3 sampling zones submitted to similar irradiation in the 3 weeks prior to sampling. Enrichment was performed on the basis of the generic GOslim ontologies for the three classes: molecular function (GOslim_MF), biological process (GOslim_BP), and cellular component (GOslim_CC) as defined by the Gene Ontology consortium. Each protein in each GO term was weighted by the number of spectral counts divided by the number of residues. Each GOslim term was then quantified by the sum of the corresponding proteins for each sample and for each of the three genera, normalized by the sum of the GOslim terms within each GOslim class and genus. We propose to call this quantification parameter “Normalized Gene Ontology Abundance Factor” (NGOAF) in analogy to the classical quantification parameter “Normalized Spectral Abundance Factor” (NSAF) used in proteomics (Paoletti et al., 2006; Christie-Oleza et al., 2012). As shown in Supplementary Tables 3, 4, the 4 most represented GOslim terms for *Sphingomonas* molecular functions were ion binding, oxidoreductase activity, RNA binding, and structural molecule activity. For *Caulobacter*, the top four terms were ion binding, oxidoreductase activity, transmembrane transporter activity and RNA binding, and for *Acidovorax*, ion binding, transmembrane transporter activity, translation factor activity and GTPase activity in Supplementary Table 3 and ion binding, transmembrane transporter activity, oxidoreductase activity and RNA binding in Supplementary Table 4. The main molecular functions are thus rather similar for all three types of bacteria, including BWW or not. For example, GOslim_MF terms such as ion binding and oxidoreductase activity are amongst the prominent functions in all three genera with 13–18% and 7–9% of the total quantified GOslim_MF terms, respectively. Some terms are also observed in the top six GOslim_BP of all three genera, such as: biosynthetic process, transport and metabolic process of small molecules. Supplementary Figure 1 shows heatmaps based on NGOAF results with row scaling, representative of functional differences between sampling sites for both Caulobacter and Sphingomonas genera. For instance, the GO_MF term “ion binding” appears proportionally more important for *Sphingomonas* in the gamma irradiated zone compared to other sites, which is reversed for *Caulobacter*.

**FIGURE 4:**
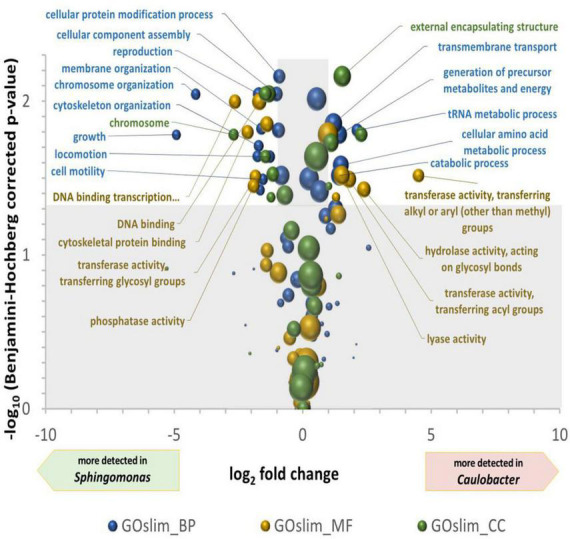
Volcano plot of the differential detection of GOslim terms between *Caulobacter* and *Sphingomonas*. The X-axis is the ln2 of the quantification of GOslim terms associated to Caulobacter divided by their quantification associated to Sphingomonas. The Y-axis is the -log10 of bilateral T-test *p*-values, after Benjamini–Hochberg multiple tests correction, comparing the four quantifications of *Caulobacter* and *Sphingomonas* GOslim terms in the four wall scrubs. The areas of the spheres are proportional to the defined quantification of the *Caulobacter* and *Sphingomonas* GOslim terms. Significantly different GO terms between genera are found in the unshaded area, i.e., with more than two-fold modulation and a –log (*p*-value) greater than 1.3.

Differences between the two most abundant genera, *Sphingomonas* and *Caulobacter*, were in-depth analyzed. A bilateral Student’s *t*-test was computed between the NGOAF for the four wall biofilms considered as replicates for the functional expression of the different genera (Supplementary Table 3) and for the three wall biofilms excluding BWW (Supplementary Table 4), yielding very similar results with decreased confidence coherent with reduced replication for similar samples. A multiple tests correction of p-values was applied using the Benjamini–Hochberg procedure. A plot of the differential expression of GO terms of the three classes is presented in Figure 4. The volcano plot shows the over-detected GO terms in *Caulobacter* on the positive values of the X-axis and in *Sphingomonas* on the negative part of the X-axis. An indication of an active growth of *Sphingomonas* can be observed by the increase in GOslim_MF terms corresponding to DNA binding and DNA binding transcription factor activities, as well as by the production and assembly of cellular components over-detected in *Sphingomonas*. DNA-related terms could also be associated with a lower DNA repair activity in *Caulobacter* compared to *Sphingomonas* under the specific pool condition. GO terms over-detected in *Caulobacter* are more indicative of metabolic degradation of molecules. Energy related terms, such as hydrolase and lyase GOslim_MF terms, carbohydrate process, precursor metabolites and energy production, and catabolic process GOslim_BP terms were over-detected compared to *Sphingomonas*. The functional differences observed could be related to the specific metabolism of *Caulobacter*. In particular, the overrepresentation of enzymes involved in energy and molecule degradation in *Caulobacter* may be linked to its oligotrophic capabilities.

## Discussion

Sampling minute amounts of precious biofilm samples from difficult environments such as a pool used to store nuclear elements offers few analytical options if taxonomical characterization and estimation of the different biomass contributions are the objectives. The extraction of the DNA present, amplification of 16S/18S rRNA gene amplicons, sequencing, and taxonomy analysis of the sequences is a first option. The reliability of this approach has been challenged as bulk DNA extraction and PCR amplification of rRNA genes are problematic because their yields can be species-dependent (Klein, 2011; Pible and Armengaud, 2015). Furthermore, the use of rRNA information alone tends to be insufficient for current taxonomy purposes, as a relatively high percentage (at least five percent) of 16S RNA sequence records are estimated to contain substantial anomalies (Ashelford et al., 2005). Another option is to sequence all the DNA extracted from the sample by metagenomics. However, this approach may necessitate an amplification step to get enough material for library construction, which could thus introduce some bias in the results. Here, we explored another option relying on peptide extraction and identification by high-resolution tandem mass spectrometry. The wealth of information obtained in terms of taxonomy appears quite interesting because peptide sequences are highly discriminative. Indeed, the information in peptides is encoded by 20 different possible amino acids. This results in highly condensed information compared to nucleic acids where the information is encoded by four different nucleotides. Here, a total of 724 bacteria-specific peptides were identified for the BNW biofilm with the equivalent of 0.05 mg of biological starting material. With an average length of 10 amino acids per peptide, these peptides are totaling 7,240 amino acid positions. Because two amino acids (leucine and isoleucine) are indistinguishable by tandem mass spectrometry, the theoretical combinations of all amino acids lead to an impressive number of combinations (19^7240^), which can theoretically be obtained by sequencing the equivalent of a nucleic acid sequence totaling 42 trillions of nucleotide positions. However, due to the history of Life starting from a common ancestor, the molecular relationships between organisms and the constraints of protein folding for stable structure and function, the sequences possibilities are more limited than this theoretical estimation. Anyway, the taxonomic identification based on the biofilm dataset collected in the present study is highly reliable because it is based on a large number of taxon-specific peptides. As previously demonstrated, metaproteomics and metagenomics deliver an identical taxonomic picture of complex samples because of the high density of molecular information analyzed (Jouffret et al., 2021). When comparing tandem mass spectrometry proteotyping and nucleic acid-based approaches, the amount of material needed is roughly the same order of magnitude. However, due to nucleic acids amplicon amplification or metagenome sequencing and its inherent biases, the presence of contaminants may be exacerbated while no bias is expected for the proteotyping methodology. In addition, the approach based on proteins is significantly faster, as the time to results could be less than 5 h as previously reported (Armengaud, 2023), and hence the workload is significantly reduced.

Here, we have shown that phylopeptidomics can help to establish the composition of the communities of microorganism present in biofilms and to estimate their contribution to biomass directly through their peptide signal. Metaproteomics is more accurate for biomass estimation than DNA sequencing methods, as has recently been established with protein-centric (Kleiner et al., 2017) or peptide-centric (Pible et al., 2020) methodologies. Here our methodology differs significantly from the classical metaproteomics protein-centric strategy (Heyer et al., 2015; Kleiner et al., 2017; Muth et al., 2018). Indeed, here the peptidome signal is directly mapped to taxonomic information while more classical metaproteomics approaches rely on protein inference and then assigning the detected proteins to taxonomic information. Protein inference is the procedure to assemble identified peptide sequences into the most appropriate set of proteins. This inference is far from trivial when multiple organisms share a large number of proteins with partially similar sequences (Armengaud, 2023). By bypassing the protein inference problem, the peptide-centric approach provides a more direct and unbiased view of the taxonomical units present in the sample, and thus a better estimation of their contributions to the biomass (Pible et al., 2020). This said, the limits of our approach can be discussed hereafter. First, the current lack of the NCBInr database to comprehensively represent the biodiversity on Earth is trivial. The method may not be applicable to identify new microorganisms from branches of Life whose genome has not yet been sequenced, or to accurately provide the most exact taxonomic rank (species, subspecies, or strains ranks). Currently, the generous efforts of the scientific community to sequence the complete genome of pathogens, emergent pathogens and environmental samples are helping to fill this gap over time. Consequently, it is expected that the peptide-centric method proposed in the present study become more powerful and efficient in the future with further database updates. A second caveat relates to the current detection limit of tandem mass spectrometers and the impossibility to amplify the peptide signal as in nucleic acid polymerase chain reaction. However, we have shown here that an amount of approximately 0.02 mg of biological starting material was sufficient to identify the three main microbial components of the TNW biofilm, as well as their respective contributions to the biomass using a Q-Exactive HF tandem mass spectrometer. For sure, this current sensitivity limit may be lowered with newer tandem mass spectrometers as suppliers tend to regularly increase the sensitivity and acquisition speed of their instruments. These limitations have to be balanced against the analytical speed and cost achieved by our methodology. In the present study actually, only 60 min of tandem mass measurements were needed per sample and data interpretation was straightforward.

To directly sample biofilms from aquatic environments without introducing any specific support, we proposed the use of a Spontex scraping sponge which, as shown here, is able to scrub the biofilm and trap part of it, thus allowing its recovery. The small amount of material obtained, even at 4 m depth in the pool, was sufficient for its analysis by metaproteomics. The original technique implemented here to collect biofilms can be used remotely more easily than the techniques described so far to investigate hostile environments.

The three genera observed in these samples, *Sphingomonas*, *Caulobacter*, and *Acidovorax*, are all environmental bacteria. The genus *Sphingomonas* is known as largely ubiquitous. Representatives of this genus have been found in different environments, such as marine and freshwater environments, soils, and deep subsurface (Cavicchioli et al., 1999; Armengaud et al., 2000; Ryan and Adley, 2010). Noteworthy, bacteria of the *Sphingomonas* genus have already been identified in spent nuclear fuel pools from the Cofrentes power plant, Valencia, Spain (Chicote et al., 2005), in Brazil (Silva et al., 2018), and in the Idaho Nuclear Technology and Engineering Center on the INL site, USA (Bruhn et al., 2009). It is difficult to compare the radiation doses received by microorganisms between different facilities. In spent fuel cooling pools, microorganisms are exposed to doses ranging from high to zero depending on the distance to the fuel elements and the time elapsed since the removal of the fuel from the reactor core. In the experiment carried out by Bruhn et al. (2009), a mixture of bacteria isolated from fuel cooling pools of the INL site was irradiated at about 2 Gy/h, which corresponds to the dose rate emitted by the spent fuel after a cooling period of approximately a year or more after removal from the core. Although *Sphingomonas* bacteria were introduced into the initial mixture, they were not reported in the biofilms formed under irradiation or in the liquid after a total irradiation dose of 570 Gy. In the study by Silva et al. (2018), it was assumed that the microorganisms identified had passed through the 12 m water column of the pool where the spent fuel elements were stored, emitting a dose rate of 0.4 Gy/h. *Sphingomonas* bacteria identified in our study were able to survive and even proliferate with active metabolism under irradiation condition. Some *Sphingomonas* are known to be multi-resistant but are not considered as extremophiles *per se*. The genome of *Sphingomonas* strain AntH11 isolated from the Antarctic Dry Valleys comprises numerous stress response genes (116 in total) for facing the extreme environmental conditions encountered in Antarctica, i.e., hyper aridity, UV radiation and cold temperatures (Gunnigle et al., 2015). Another strain of *Sphingomonas* exhibited high resistance to UV radiation with a 10% survival at a 1,500 J m^–2^ UV dose (Joux et al., 1999). Finally, *Sphingomonas wittichii* RW1 has been well characterized as encompassing an impressive enzymatic arsenal for the degradation of dibenzo-p-dioxin (Armengaud and Timmis, 1997; Armengaud et al., 1998; Hartmann and Armengaud, 2014).

The genus *Caulobacter* is also ubiquitous and can be found in aquatic environments, freshwater sediment and soils (Jin et al., 2014) and has also been found associated with plants (Edulamudi et al., 2011). Its presence in the vicinity of radionuclide-contaminated environments has been reported. The *Caulobacter* strain OR37 isolated from subsurface sediments at Oak Ridge (USA) was proven to be tolerant to 200 μM of uranium at pH 7 (Utturkar et al., 2013). The genus *Caulobacter* has been identified in spent nuclear fuel storage pools in Sellafield (UK) (Foster et al., 2020; Ruiz-Lopez et al., 2020). *Caulobacter* bacteria are well known for their distinctive ability to live in low-nutrient environments (Hu et al., 2005). Detection of their high level of ion binding and oxidoreductase activity is linked to this ability.

Bacteria belonging to the *Acidovorax* genus have been isolated from lawn soil, water, commensal flora, activated sludge plant water and have also been described as phytopathogen (Willems et al., 1990; Schulze et al., 1999; Gardan et al., 2000). Some *Acidovorax* strains can oxidize H_2_ as an energy source (Brenner et al., 2005), H_2_ being formed by water radiolysis. Members of the genus *Acidovorax* have been found in spent nuclear fuel storage pools in Brazil and in the UK (Silva et al., 2018; Foster et al., 2020). *Acidovorax* bacteria identified in the present study were probably less resistant to radiation than members of the genera *Sphingomonas* and *Caulobacter* detected, as evidenced by its absence from the BWW biofilm.

The functional analysis performed with gene ontology terms reveals striking differences between the two main microorganisms. On the one hand, *Sphingomonas* metabolism is oriented toward growth and cell division (membrane organization, chromosome organization, growth, locomotion) while on the other hand, *Caulobacter* metabolism is oriented to specific metabolic process typical of stationary phase (transmembrane transport, generation of precursor metabolites, amino acid metabolism, tRNA metabolism).

In conclusion, we have shown in this work that the microbial composition of biofilms can be directly analyzed using metaproteomics from as little as 0.02 mg of biological material. An original method for remote sampling of biofilms is presented, which can be employed to investigate other hostile environments. An innovative methodology for the processing of the MS/MS spectra data obtained after protein extraction has been developed for the proteotyping of microorganisms present in biofilms. The strategy developed here can be used to identify microorganisms present in any kind of samples and to decipher their respective contributions to the biomass. A last asset of proteomic analyses is that they provide direct access to the functioning of microorganisms present in microbial communities.

## Data availability statement

The mass spectrometry proteomics data have been deposited to the ProteomeXchange Consortium via the PRIDE partner repository with the dataset identifier PXD039499 and doi: 10.6019/PXD039499 and are publicly available (http://www.ebi.ac.uk/pride/archive/projects/PXD039499).

## Author contributions

OP, PP, CR, and JA: study conception and design and draft manuscript preparation. PP, GS, CR, and JA: data collection. All authors contributed to analysis and interpretation of results, reviewed the results, and approved the final version of the manuscript.

## Footnotes

1
http://www.matrixscience.com/msparser.html

2
https://www.ebi.ac.uk/GOA

3
http://www.geneontology.org/faq/how-do-i-map-set-annotations-high-level-go-terms-go-slim

4
http://www.geneontology.org/ontology/subsets/goslim_generic.obo

## Supplementary material

Supplementary Figure 1 Heatmaps based on NGOAF results with row scaling, representative of functional differences between sampling sites for both *Caulobacter* and *Sphingomonas* genera.
