# Supplementary figures and images for: Taxonomical composition and functional analysis of biofilms sampled from a nuclear storage pool
Source: Front Microbiol. 2023 Apr 13;14:1148976. doi: 10.3389/fmicb.2023.1148976 (PMC10133526; doi:10.3389/fmicb.2023.1148976)

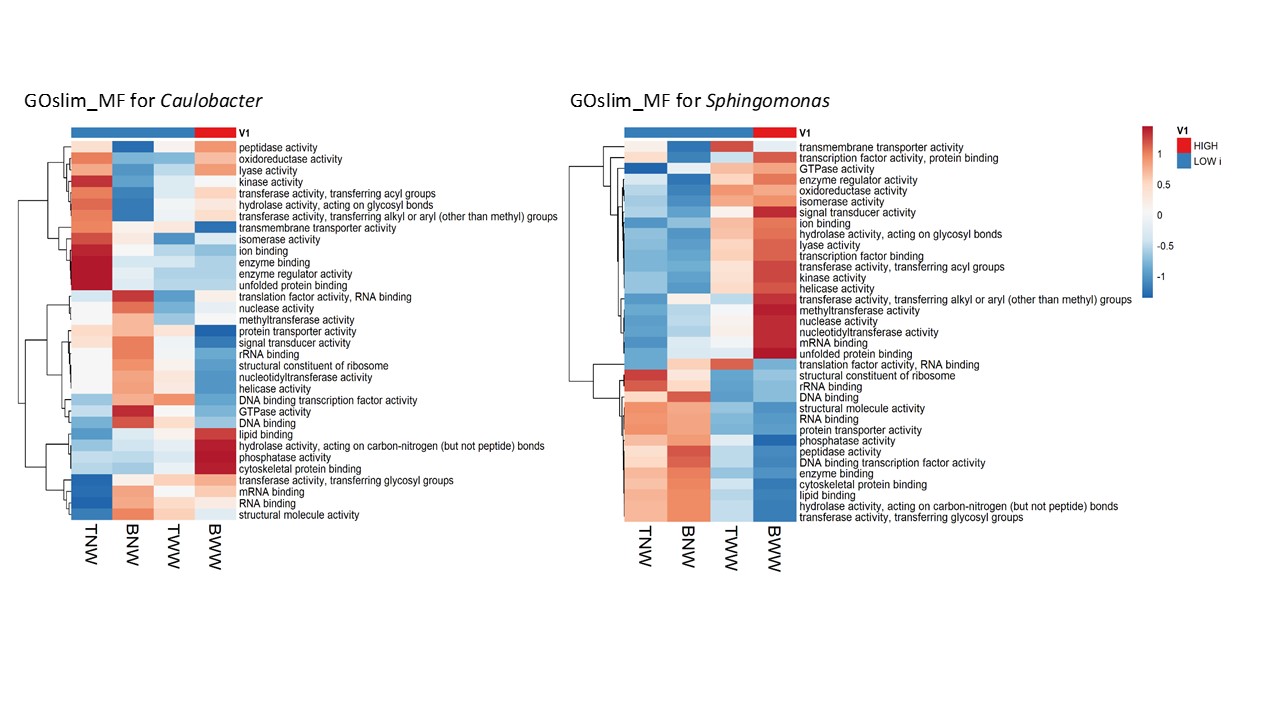

Supplement: Supplementary Figure 1 — Heatmaps based on NGOAF results with row scaling, representative of functional differences between sampling sites for both Caulobacter and Sphingomonas genera. [file Image_1.JPEG]
